# Measuring gun violence in police data sources: transitioning to NIBRS

**DOI:** 10.1186/s40621-022-00376-8

**Published:** 2022-05-02

**Authors:** Susan T. Parker

**Affiliations:** Department of Health Management and Policy, University of Michigan School of Public Health, 1415 Washington Heights, M3148 SPH II, Ann Arbor, MI 48109-2029 USA

**Keywords:** Gun violence, NIBRS, Crime, Violence, Police, Data accuracy

## Abstract

**Background:**

The majority of gun violence in the United States does not result in physical injury and therefore cannot be completely measured using hospital data. To measure the full scope of gun violence, the nation’s crime reporting systems that collect police reports of crimes committed with a firearm are vital. However, crime data reporting conventions may underestimate gun violence in the U.S. This paper compares crime data sources to assess underestimation of gun violence.

**Findings:**

The Federal Bureau of Investigation’s Summary Reporting System (SRS) and National Incident Based Reporting System (NIBRS) measures of gun violence were compared in 2019 for states comprehensively reporting data to both systems. Gun violence is underestimated in the SRS compared to NIBRS. Within the sample, 18.8% more aggravated assaults with a firearm are recorded and 2.1% more robberies with a firearm are recorded in NIBRS. The proportion of assaults and robberies committed with a firearm measured in both sources did not differ. If the additional gun violence events recorded in the NIBRS sample are consistent with national crime reporting, the number of additional gun violence events per year captured using NIBRS totals approximately 65,071 additional events, or an additional 178 gun violence events per day. Of the additional gun violence events, approximately 31% are due to omitted crime categories, with the remaining variation driven mostly by aggravated assaults with a firearm.

**Conclusions:**

Police data are important data sources for estimating the full scope of gun violence. Comparisons between police data sources suggest that the proportion of crimes committed with a firearm is unchanged. Due to crime reporting conventions, however, the number of gun violence events may be substantially understated. Despite advantages in measuring gun violence, agency participation in NIBRS is alarmingly low and jeopardizes accurate and reliable national crime data.

## Introduction

In the United States, 21,570 people were the victims of homicide by gunshot wound in 2020, constituting 59 deaths each day (WISQARS Nonfatal Injury Reports 2019; Expanded Homicide 2021). While homicide data is comprehensive, no comprehensive data source currently measures all gun violence victimization (Roman 2020). Gun violence victimizations occur when a victim is threatened or attacked with a firearm. Personal threats or attacks with a firearm are aggravated assaults and threats to a victim’s property are robberies, though an element of assault is present in robberies (Summary Reporting System User Manual-FBI 2013). Gun violence victimization therefore includes: assaults and robberies where a firearm is used to threaten violence but is not discharged; assaults and robberies where a firearm is discharged but no injuries occur; and assaults and robberies where a firearm is discharged and victim(s) suffer gunshot wounds. Both police and hospital records record gun violence victimization. Police data sources measure reported gun crimes, consisting of gun assaults and robberies, and hospital data sources measure gunshot wounds, recording whether the wound occurred in the course of a gun crime.

Hospital data captures the physical toll of gunshot wounds, which is both substantial and costly (Gani et al. 2017; Cook and Ludwig 2000). But gun violence victimization includes additional violence not captured by measures of morbidity and mortality. In fact, gun violence not resulting in physical injury constitutes the majority of gun violence in the U.S. Assaults and robberies committed with a firearm recorded in police data annually affect approximately 300,000 people (Crime in the U.S. 2019). In contrast, in 2019 approximately 50,000 victims annually were non fatally shot by another person in a criminal assault or robbery and were treated for their injury (Fowler et al. 2015). The number of gun violence victims varies substantially when victimization is assessed using police data sources compared to hospital data sources. The majority of gun assaults and robberies do not result in physical injuries treated in a hospital and measuring gunshot wounds alone understates the full scope of harms caused by gun violence. While police data do not comprehensively measure gunshot wounds, police measures of gun violence are an essential data source for the majority of gun assaults.

To measure the full scope of gun violence, the nation’s crime reporting systems that gather police data play an invaluable role. The Federal Bureau of Investigation’s Uniform Crime Report (UCR) Summary Reporting System (SRS) served as the nation’s measure of crime since the 1930s. As of January 1, 2021, the FBI opted to retire the UCR SRS program for its successor crime reporting program, the National Incident-Based Reporting System (NIBRS) (Hanson 2021). Both crime reporting systems record assaults with a firearm regardless of whether the firearm is discharged or not, or the gunshot assault wounds the intended target or not.

For decades, however, reporting conventions in the UCR SRS have limited the precision and detail with which gun violence is measured (Strom and Smith 2017; Hanson 2021). First, guns used in violent crime were only recorded in relation to aggravated assaults and robberies, not the full scope of gun violence. Other crimes, such as sexual assaults, where a firearm may be used are either not recorded in the SRS or the option to include a weapon is not available (Summary Reporting System User Manual-FBI 2013). Second, to conveniently summarize a crime incident, only the most serious crime that transpired in an event was recorded (Hanson 2021). This convention, known as the “hierarchy rule,” results in underreporting of aggravated assaults with a gun. Specifically, in cases where a robbery and an aggravated assault occurred during the same crime event, only the robbery would be counted. Finally, a crime incident affecting multiple victims was counted as a single incident. In addition, the SRS does not record any information on injury or firearm discharge in the course of a gun assault or robbery.

NIBRS improves some aspects of gun violence measurement compared to the legacy UCR SRS data collection, but to date, no study has assessed the extent to which the nation’s new crime reporting system more precisely measures gun violence. Using data from both the SRS and NIBRS, this paper compares how the two sources differ in measurement of gun violence incidence and victimization. This comparison is timely as police agencies adopt NIBRS reporting standards by 2021, constituting the most substantial change crime data reporting has undergone in 9 decades.

## Methods

Data from the nation’s two crime reporting systems, the UCR SRS and NIBRS, were compiled from a sample of states and law enforcement agencies that reported complete, comprehensive crime data to both systems in 2019, the most recent year of available data.

### Summary Reporting System (SRS)

The FBI’s Uniform Crime Reporting (UCR) Program Summary Reporting System (SRS) has for decades gathered summary counts of crime reported to law enforcement agencies in the U.S.. The SRS gathers summary counts of crimes including violent crimes, such as rape, robbery, and aggravated assault, and property crimes. In 2019, the SRS included over 18,000 law enforcement agencies nationwide that typically cover more than 90 percent of the U.S. population (Maltz 2006; FBI 2021). The final year of SRS data collection by the FBI was 2020.

The SRS has a series of limitations that may result in undercounting of gun violence reported to law enforcement agencies. The SRS records the type of weapon for only three crime types: robbery, aggravated assault, and murder. For all other types of crime, the SRS either does not specify weapon type or does not include the crime category. For instance, the SRS includes rape but does not provide a breakdown by weapon type. Other crimes that may be committed with a firearm, such as sexual assault or kidnapping, are not included in the SRS (National Incident-Based Reporting System Technical Specification 2020). Further, the SRS records only the single most serious offense associated with each crime, a reporting convention known as the “hierarchy rule” (Federal Bureau of Investigation 2021).

UCR SRS data used in this study were obtained from Jacob Kaplan’s Open ICPSR repository (Jacob Kaplan 2021).

### National Incident Based Reporting System (NIBRS)

Development of the National Incident Based Reporting System (NIBRS) commenced in the 1980s with the intention of improving on SRS limitations (National Academies of Sciences, Engineering, and Medicine 2018). NIBRS records offense types that the SRS omits, gathering incident-level data on 52 offenses (Federal Bureau of Investigation 2021). NIBRS also allows a firearm to be reported with more offense types than in the SRS, including sexual assault, rape, kidnapping, and extortion. Further, NIBRS does not impose a hierarchy rule and counts each of the crimes committed in any one incident, and specifies the number of victims in that incident. While NIBRS does measure victim injuries, no field exists for gunshot injuries making determination of a firearm injury impossible.

Starting in 2021, the FBI records police data in NIBRS format only, with substantial reductions in population coverage. As of 2019, NIBRS covers only 53% of the U.S. population (FBI Crime Data Explorer 2020).

NIBRS data were obtained from the FBI’s Crime Data Explorer, which features tables counting incidents, offenses, and victims. Incidents count the number of distinct incidents of a crime that occur and allow for a crime incident to be classified as multiple types of crimes. The victim tables expand on the crime incident to link multiple victims to a crime incident.

### Sample selection

To compare UCR SRS and NIBRS measurements of gun violence, states whose population coverage in NIBRS is at or above 98% population coverage from 2015–2019 statewide were selected. These states include Colorado, Kentucky, Michigan, South Carolina, Tennessee and Virginia and comprise approximately 11% of the U.S. population. Law enforcement agencies within each state were then matched between UCR SRS and NIBRS sources to compare measures across the sources. All measures are calculated using data from 2019, the most recent year of data available both from the SRS and NIBRS.

### Measures

Several measures were constructed from incident-level NIBRS data to be directly comparable to aggregated UCR SRS data (Law Enforcement Support Section (LESS) Crime Statistics Management Unit (CSMU) 2013). The UCR SRS reports a robbery incident count and aggravated assault as a victim count. NIBRS data were adjusted to match SRS conventions.

To calculate the number and proportion of crimes committed with a firearm, NIBRS weapons categories including handgun, rifle, shotgun, other firearm, and automatic firearm categories were counted if they were attributed to a crime incident for robberies, or a crime victim or victims for aggravated assault. The corresponding UCR SRS measure is used for comparison. The rates of assaults and robberies committed with a firearm are compared by calculating the proportion of robbery and assault committed with a firearm using the denominator of total robberies and assaults. To identify multiple offenses in NIBRS, the unique incident number of each firearm crime was matched to all other offenses associated with the same incident identifier.

To measure additional firearm crime captured in NIBRS, two categories were constructed. NIBRS crime categories with a firearm include kidnapping, extortion, rape, sexual assault, and weapons law offenses in addition to aggravated assault and robbery that both NIBRS and SRS both capture. However, in NIBRS, many of the additional firearm categories are not frequently populated. For this reason, rape and sexual assaults are combined into a single category and kidnapping is combined with exhtortion to form an additional single cateogry.

Comparisons are made at the state unit of analysis as well as the FBI’s population group variable, which is a categorical classifier describing the size and nature of the population each law enforcement agency serves. Population groups under 100,000 residents are combined into one measure as they are less frequent than large city reporting and have similar reporting patterns.

### Analysis

NIBRS and SRS count and proportion measures of robbery and aggravated assault with a firearm are compared to understand to what extent the SRS may undercount gun violence in 2019, the most recent year of available NIBRS data. Proportions of robbery and aggravated assault with a firearm are calculated in reference to a denominator of all aggravated assaults and robberies, which may be committed without a firearm or with another weapon, such as a knife. These proportions are compared using chi-squared tests of proportion to assess whether the measures differ statistically at the 0.05 level of significance.

Because the SRS suppresses counts of crime when a more severe crime also occurs according to the hierarchy rule, the SRS may underestimate gun violence. To assess the extent of variation from this reporting convention, offenses that would have been suppressed in the SRS are tabulated.

To assess agency coding, the proportion of crimes involving firearms by source are compared among agencies serving a population larger than 100,000. If the distribution shows a large proportion of agencies with exactly no difference between their SRS and NIBRS reports, agencies may not have fully adopted reporting multiple crimes per incident. Similarly, if only a handful of agencies account for the majority of the NIBRS and SRS difference, the problem may be individual agency NIBRS adoption. If agencies on average tend to report additional offenses with stable proportions of firearm use, as agencies have experience reporting weapons for these crimes for decades, this is suggestive that NIBRS may improve existing coding of offenses. Law enforcement agency population groups are also used to assess if crime reporting differs by population type and size.

Next, categories of gun violence that are not measured in the SRS system but are tracked in NIBRS are tabulated to understand the extent to which underestimation of gun violence in the SRS may be a function of more limited SRS crime categories.

Analysis was performed in R 4.1.0. This study was considered exempt from review by the institutional review board at the University of Michigan.

## Results

Nationwide, 27.6% of aggravated assaults and 36.4% of robberies were committed with a firearm in 2019 (Table 1). In contrast, 38.1% of aggravated assaults and 50.7% of robberies were committed with a firearm in 2019 in the sample of states that report to NIBRS (Table 2).

Within the sample, NIBRS recorded greater counts of gun violence events compared to the SRS, but the proportion of violence involving a firearm does not differ between the two sources (Table 2). Compared to the SRS, NIBRS recorded higher counts of both aggravated assaults and robberies committed with a firearm. The most substantial difference was driven by aggravated assaults. NIBRS captured on average 22.3% more aggravated assaults with a firearm in the sample (Table 2). For all states with the exception of Colorado and Kentucky, the proportion of aggravated assault with a firearm in NIBRS is statistically higher than the proportion reported in the SRS. Robberies with a firearm differ less substantially, with NIBRS recording only 2.6% more robberies committed with a firearm than the SRS (Table 2).

**Table 1:** Firearm crimes recorded in the SRS, nationwide (2019)

Nationwide	SRS	
	*n*	%
Aggravated assault with firearm	226,646	27.6
Rape	139,815	–
Robbery with firearm	97,548	36.4
Homicide with firearm	12,105	73.7

**Table 2:** Firearm crimes recorded in the SRS versus NIBRS by state (2019)

	SRS		NIBRS		Difference (NIBRS vs SRS)		*p*-value
	*n*	%	*n*	%	*n*	%	
*Aggravated assault with firearm*							
Colorado	4173	31.8	5009	31.5	836	20.0	0.50
Kentucky	2162	34.0	2824	36.6	662	30.6	0.21
Michigan	8925	27.3	9893	31.0	968	10.8	0.05
South Carolina	9136	41.4	10,869	44.7	1733	19.0	0.00
Tennessee	13,921	35.4	17,654	43.5	3733	26.8	0.00
Virginia	3578	31.5	4976	35.3	1398	39.1	0.00
Total	41,895	38.1	51,225	38.1	9330	22.3	0.80
*Robbery with firearm*							
Colorado	1315	38.6	1337	38.0	22	1.7	0.62
Kentucky	1008	46.7	1037	45.0	29	2.9	0.27
Michigan	2253	43.4	2265	43.4	12	0.5	0.90
South Carolina	1829	57.1	1882	55.9	53	2.9	0.34
Tennessee	3798	61.8	3923	60.0	125	3.3	0.04
Virginia	1771	50.3	1807	49.2	36	2.0	0.39
Total	11,974	50.7	12,251	49.8	277	2.3	0.05

Table 3 reports approximately 16% of the difference in aggravated assaults is due to the hierarchy rule and 33% of the difference in firearm robberies is attributable to the hierarchy rule. For aggravated assaults, one agency accounted for a large proportion of the difference in aggravated assaults with a firearm, reporting 1,837 additional aggravated assaults with a firearm (Metropolitan Nashville Police Department (MNPD)).

**Table 3:** Proportion of SRS versus NIBRS difference by cause

Aggravated assault with firearm	*n*	% of NIBRS versus SRS difference
Total in NIBRS	51,225	
Total in SRS	41,895	
Difference	9330	
*Hierarchy rule application*		
Murder	879	9.4
Rape	6	0.1
Robbery	643	6.9
Largest reporting agency	1837	19.7
Total	3365	36.1

Robbery with firearm	*n*	%
Total in NIBRS	12,251	
Total in SRS	11,974	
Difference	277	
*Hierarchy rule application*		
Murder	58	20.9
Rape	32	11.55
Total	90	32.5

**Fig. 1:**
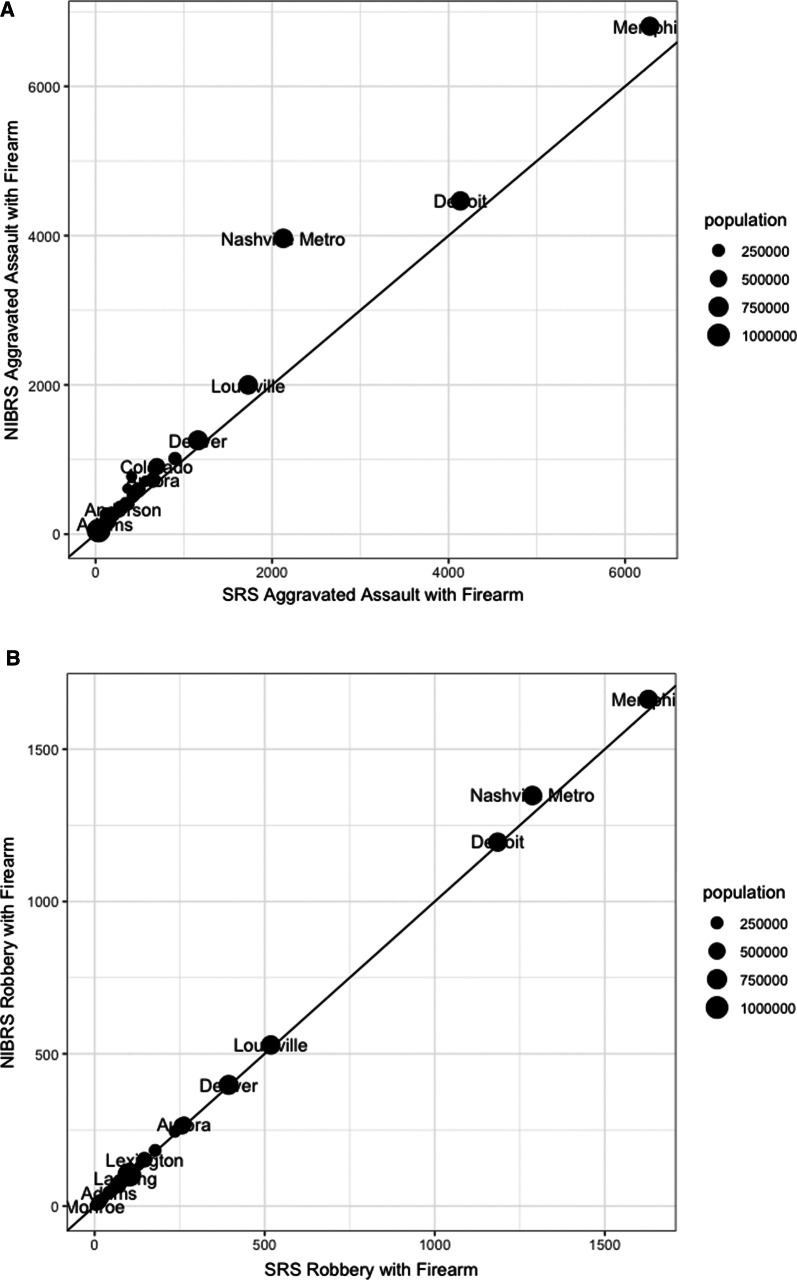
SRS versus NIBRS large agency-level reporting of firearm assault, robbery (2019). Panel **A**: aggravated assault with firearm. Panel **B**: robbery with firearm

To understand agency-level reporting, Fig. 1 compares counts of firearm assaults and robberies among agencies serving populations larger than 100,000. If the proportion of robberies and assaults committed with a firearm are the same in the SRS and NIBRS, the agency’s difference will be approximately zero and will adhere to the reference line. For aggravated assault with a firearm in Panel A, agencies that report more aggravated assaults tend to report more NIBRS incidents. MNPD’s increase is the largest among sample agencies. Panel B depicting robbery with a firearm counts do not differ by agency.

**Fig. 2:**
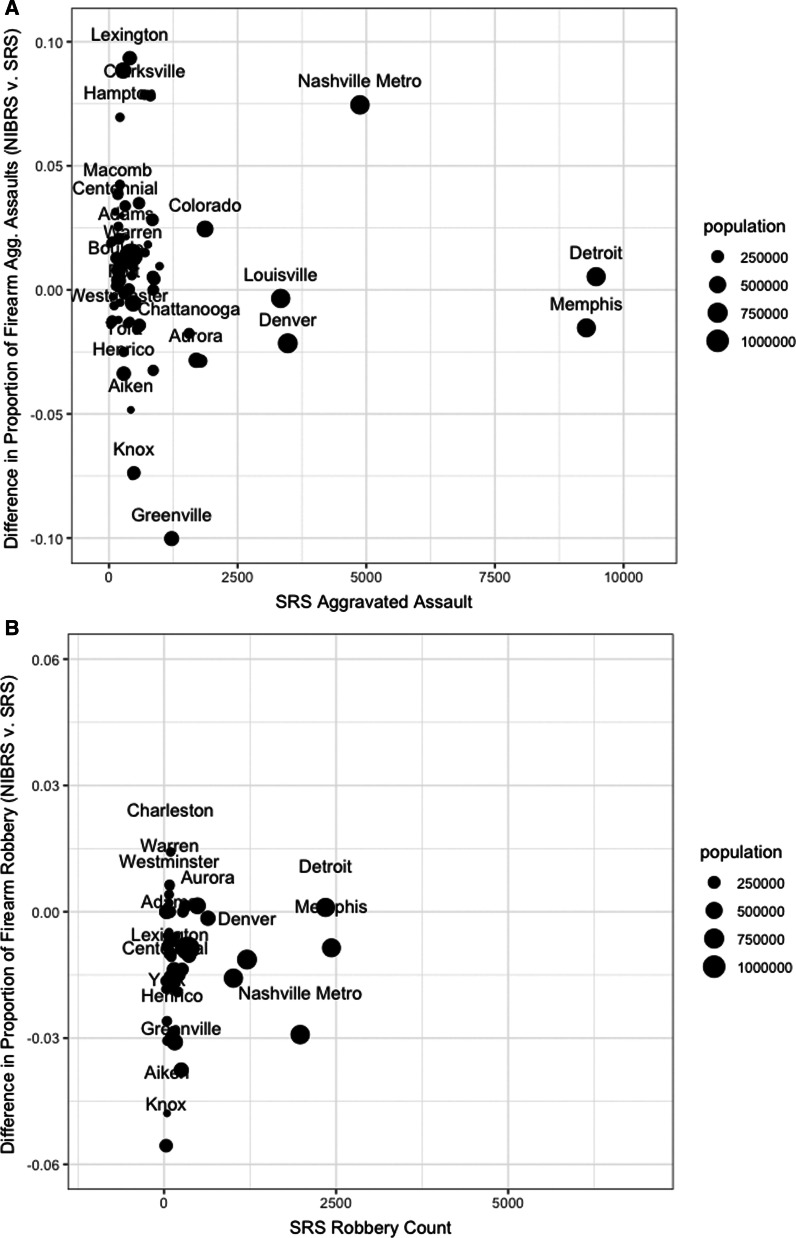
Proportion differences in large agency-level reporting of firearm assault, robbery (2019). Panel **A**: aggravated assault. Panel **B**: robbery

Figure 2 assesses whether the proportion of aggravated assaults and robberies committed with a firearm differ by source. Panel A shows some agencies cluster at positive increases of less than 3% in recording of firearm assault in NIBRS, which is consistent with coding improvements identifying additional firearm assaults. However, Fig. 2 also makes clear that some agencies record more aggravated assaults and robberies without a firearm, resulting in the reductions in the rate of firearm robbery and firearm aggravated assault. The magnitude of the change in the proportion of assaults and robberies committed with a firearm are substantial for some agencies.

Table 4 reports the difference in proportions of firearm assaults and robberies in the SRS and NIBRS for agencies that report at least both an assault and robbery with a firearm. Agencies categorized as “no difference” report equal firearm assault and robbery proportions by source. Among the 266 agencies that do so for aggravated assault with a firearm, 255 report the exact same number of robberies with a firearm to both NIBRS and the SRS suggesting that multiple offense coding may not be fully adopted. Table 5 examines whether differences in reporting overall assaults or robberies may drive differences across agency population, suggesting no clear trend by population served.

**Table 4:** Differences in SRS versus NIBRS proportions of firearm assault, firearm robbery among reporting agencies (2019)

	*n*	%
*Aggravated assault with firearm*		
Less than -5%	71	7.5
Less than 0 to -5%	280	29.6
No difference in reporting	266	28.1
Greater than 0 to 5%	235	24.8
Greater than 5%	94	9.9
Agency total	946	100.0
*Robbery with firearm*		
Greater than -5%	54	5.7
Less than 0 to -5%	107	11.3
No difference in reporting	745	78.8
Greater than 0 to 5%	26	2.7
Greater than 5%	14	1.5
Agency total	946	100.0

**Table 5:** Firearm crimes in SRS versus NIBRS by agency population group (2019)

Population Group	Aggravated assaults			Aggravated assaults with firearm		
	SRS	NIBRS	Percent difference (%)	SRS	NIBRS	Percent difference (%)
City 500,000 thru 999,999	30,432	36,059	15.6	15,431	18,492	16.6
City 250,000 thru 499,999	4242	5329	20.4	1504	1969	23.6
City 100,000 thru 249,999	13,612	16,793	18.9	4903	6427	23.7
msa-county 100,000 +	12,367	15,625	20.9	4620	5518	16.3
msa-county, non-msa, city less than 100,000	49,177	60,714	19.0	15,437	18,819	18.0
Total	109,830	134,520	18.4	41,895	51,225	18.2

	Robbery			Robbery with firearm		
	SRS	NIBRS	Percent difference (%)	SRS	NIBRS	Percent difference (%)
City 500,000 thru 999,999	8969	9371	4.3	5012	5134	2.4
City 250,000 thru 499,999	1681	1731	2.9	766	781	1.9
City 100,000 thru 249,999	3488	3603	3.2	1773	1810	2.0
msa-county 100,000 +	2471	2605	5.1	1203	1232	2.4
msa-county, non-msa, city less than 100,000	7025	7310	3.9	3220	3294	2.2
Total	23,634	24,620	4.0	11,974	12,251	2.3

Crimes not reported to the SRS but recorded in NIBRS constitute approximately 10% (5245/53,869 (41,895 gun assaults + 11,974 gun robberies) more instances of gun violence than are captured in the SRS measures (Table 3). In 2019, 14% of rapes and sexual assaults were committed with a firearm and 30% of blackmail and extortion crimes were committed with a firearm (Table 6).

**Table 6:** NIBRS crimes committed with a firearm (2019)

	*n*	%
*Rape and sexual assault with firearm*		
Colorado	552	19.2
Kentucky	29	2.4
Michigan	653	13.2
South Carolina	248	12.5
Tennessee	278	11.5
Virginia	529	23.4
Total	2289	14.6
*Extortion and kidnapping with firearm*		
Colorado	622	26.2
Kentucky	208	19.4
Michigan	494	44.1
South Carolina	407	35.1
Tennessee	587	28.1
Virginia	638	29.4
Total	2956	29.6
*Total NIBRS additional firearm crimes*	5245	20.5

Using the proportion of additional NIBRS gun violence incidents in the sample, an approximate calculation to estimate the national number of gun violence events is possible. For the states included in the sample, omitting MNPD for this calculation, the undercount of aggravated assaults was 18.8%; the undercount of robberies was 2.1% and the rate of rapes occuring with a firearm was 14.6%. It is not possible in the scope of this paper to ascertain whether these percentages also apply to other states. However, if these percentages are approximately correct, the implication is an additional 65,071 gun violence incidents annually not captured in the SRS. Specifically, the national number of aggravated assaults with a firearm would be 18.8% higher or total 269,255.7 (1.188 * 226,646) (Table 2). The national number of robberies with a firearm would be 2.1% higher or number 99,596.1 (1.021*97,548) (Table 2). The national number of rapes with a firearm would be the NIBRS rate of rape with a firearm, 14.6%, applied to the 2019 rape total events in the SRS, comprising a previously unreported 20,413 gun violence events (Table 2).

Taken together, the total number of additional gun violence incidents totals approximately 178 additional gun violence events per day not captured in the SRS. Of the additional gun violence incidents not captured in the SRS, approximately 31% (20,413 rapes with a firearm out of the total 65,071 additional events) are due to crime categories omitted from the SRS, in this case, rape. The remaining 69% of the additional gun violence events are due to reporting conventions in the SRS and coding changes in NIBRS.

## Discussion

This study compares the FBI’s crime reporting systems to assess the extent to which they accurately estimate gun violence. The SRS underestimates gun violence incidence in the U.S. compared to NIBRS. Underestimation of gun violence is comprised of crime types that are not recorded in the SRS or that do not allow recording of a firearm use with the crime type.

Contrary to prior studies, underestimation of gun violence in the SRS may be substantial. Partly, underestimation is driven by differences in how NIBRS records crimes compared to  the SRS. As well,  NIBRS records firearms used with additional crimes. In particular, the difference in measuring gun violence is primarily due to underreporting of aggravated assaults with a firearm in the SRS. While the hierarchy rule suppression contributes to the underreporting, it is not the primary source of the increase in NIBRS aggravated assaults with a firearm.

The systematic error and quality checks that NIBRS implements may account for a greater proportion of SRS underreporting. NIBRS data quality checks do not permit entry of conflicting crime types, and mandate recording of weapon and injury types when applicable crime types are selected (National Incident-Based Reporting System Technical Specification 2020). For instance, if an aggravated assault is recorded with no injury and no weapons, NIBRS will flag this entry for review. While the agency data suggest that some may not have fully transitioned to reporting multiple offenses per incident as the number of identical reports to the SRS and NIBRS indicates, generally, larger agencies with more resources to report quality data tended to report additional aggravated assaults with a firearm which supports the potential for coding improvement.

While NIBRS may address problems with undercounting gun violence, adoption among criminal justice agencies is very low. Despite limited adoption of NIBRS, 2020 is the last year in which the FBI will gather SRS data. While the SRS measures of gun violence are imprecise and underreported, NIBRS lacks nationwide reporting coverage.

This study has several limitations. First, this study is not nationally representative as all states do not report data to NIBRS. The states in the sample comprise a greater proportion of firearm crimes than the proportion of the U.S. population it captures, and so may reflect other factors that increase firearm crime. Second, this study relies on gun crimes reported to police and does not include crime unreported to law enforcement. Third, combining crime categories in NIBRS was necessary to minimize sparse gun violence categories. This may reflect law enforcement transition from summary reporting conventions rather than crime events.

## Conclusion

This study assesses how gun violence is measured in police data in the United States. Police data are important data sources for estimating the full scope of gun violence. The proportion of crimes committed with a firearm does not differ between the nation’s two crime reporting systems, the outgoing SRS and the new national standard NIBRS. Due to reporting conventions, NIBRS data suggests that gun violence may be understated by approximately 65,071 events per year. The majority of the gun violence not fully measured is comprised of aggravated assaults with a firearm (69%) and previosly unmeasured rape with a firearm (31%). Not reporting data to NIBRS on the part of many states is an alarming barrier to accurate and comprehensive measurement of crime in the U.S. 

## Data Availability

The datasets analysed during the current study are available in the FBI data repository, https://crime-data-explorer.fr.cloud.gov/pages/home

## Abbreviations

FBI: Federal Bureau of Investigation
UCR: Uniform Crime Reporting
SRS: Summary Reporting System
NIBRS: National Incident Based Reporting System
